# Exploring the Chemical Composition of Bulgarian Lavender Absolute (*Lavandula Angustifolia* Mill.) by GC/MS and GC-FID †

**DOI:** 10.3390/plants11223150

**Published:** 2022-11-17

**Authors:** Daniela Nedeltcheva-Antonova, Kamelia Gechovska, Stanislav Bozhanov, Liudmil Antonov

**Affiliations:** 1 Institute of Organic Chemistry with Centre of Phytochemistry, Bulgarian Academy of Sciences, 1113 Sofia, Bulgaria; 2 Faculty of Pharmacy, Medical University of Sofia, 1000 Sofia, Bulgaria; 3 Institute of Electronics, Bulgarian Academy of Sciences, 1784 Sofia, Bulgaria

**Keywords:** *Lavandula angustifolia* Mill., lavender absolute, GC/MS, chemical profiling, aromatherapy

## Abstract

Lavender (*L. angustifolia* Mill.) is an important essential oil-bearing and medicinal plant with high commercial value. Lavender scent components can be derived not only as an essential oil but also as lavender concrete or absolute. The development of reliable analytical methods for origin assessment and quality assurance is of significant fundamental importance and high practical interest. Therefore, a comprehensive chemical profiling of seven industrial samples of Bulgarian lavender absolute (*L. angustifolia* Mill.) was performed by means of gas chromatography–mass spectrometry (GC/MS) and gas chromatography with flame ionization detection (GC-FID). As a result, 111 individual compounds were identified by GC/MS, and their quantitative content was simultaneously determined by GC-FID, representing 94.28–97.43% of the total contents of the lavender absolute. According to our results, the main constituents of lavender absolute (LA) are representatives of the terpene compounds (with the dominating presence of oxygenated monoterpenes, 52.83–80.55%), followed by sesquiterpenes (7.80–15.21%) and triterpenoids (as minor components). Coumarins in various amounts (1.79–14.73%) and aliphatic compounds (hydrocarbons, ketones, esters, etc.) are found, as well. The acyclic monoterpene linalool is the main terpene alcohol and, together with its ester linalyl acetate, are the two main constituents in the LAs. Linalool was found in concentrations of 27.33–38.24% in the LA1-LA6 samples and 20.74% in the LA7 samples. The amount of linalyl acetate was in the range of 26.58 to 37.39% in the LA1–LA6 samples, while, surprisingly, it was not observed in LA7. This study shows that the chemical profile of the studied LAs is close to the lavender essential oil (LO), fulfilling most of the requirements of the International Standard ISO 3515:2002.

## 1. Introduction

Lavender (*Lavandula angustifolia* Mill.), also known as English lavender or true lavender, is a perennial sub-shrub plant belonging to the Lamiaceae family. The genus Lavandula, a rich source of phytochemicals [1,2], includes more than 40 flowering species, but only a few of them are industrially cultivated, mainly for the manufacturing of essential oil. Among them, lavender (*L. angustifolia* Mill.), spike lavender (*L. latifolia* L.) and lavandin (*Lavandula × intermedia Emeric* ex *Loisel*) are the most important.

Various species of the genus Lavandula have been used for centuries as aromatic and medicinal plants. The essential oil from Lavandula species has been known to have a wide range of biological activities, including antimicrobial, anxiolytic, anti-inflammatory activity and antioxidant properties. *L. angustifolia* is used in pharmacy, phytotherapy and aromatherapy as one of the most popular herbal remedies to treat central nervous system disorders, such as anxiety, stress, depression and sleep disorders [3,4,5]. Lavender essential oil has been found to be active against many bacteria, predominantly against Gram-positive, but also against Gram-negative bacteria, including multidrug-resistant bacteria [6]. Lavender oil has also been reported to have antifungal activity against fungi of both medical and agricultural importance [7]. It is worth mentioning that, according to the U.S. Food and Drug Administration, *L. angustifolia* has been classified as safe and has been included on the safe substances list, commonly known as “Generally Recognised as Safe” (GRAS) [8].

Lavender essential oil, due to its nice fragrance and valuable biological activity, has a wide range of applications in various industrial products, such as perfumes, pharmaceuticals, cosmetics and personal care products [7,9], as a food flavoring and preservation agent [10], in aromatherapy, etc.

Lavender, native to the Mediterranean region, is currently cultivated worldwide, but Bulgaria, the UK and France dominate lavender essential oil production. Bulgaria has a long tradition of lavender cultivation (introduced in the early twentieth century) and essential oil production, which has been quickly growing during the last decade [5]. Industrial cultivation in Bulgaria is based exclusively on true lavender (*L. angustifolia* sp. *angustifolia*), while lavandin is grown in limited areas without economic significance.

Lavender essential oil is considered of higher quality in comparison to oils from other Lavandula species. The compositions of lavender inflorescence volatiles and distilled essential oil are significantly influenced by a number of factors, such as geographical origin, genotype, the stage of inflorescence development, environmental conditions, day-time period of harvesting, flower storage and processing, etc. [11,12,13,14,15,16,17,18,19,20]. Currently, lavender essential oil is primarily produced from lavender inflorescences by conventional extraction methods, namely hydrodistillation, steam distillation, solvent extraction, and recently, using alternative technologies based on supercritical fluid extraction. The chemical composition of and production technology for lavender essential oil are under regulation by the International Organisation for Standardization (ISO 3515:2002: The oil obtained by steam distillation of recently picked lavender flowers (*L. angustifolia* P. Miller)) [12].

In addition, the valuable lavender aroma can be extracted as a wax-like substance called concrete by non-polar solvent extraction (usually with petroleum ether), which, followed by ethanol re-extraction, produces lavender absolute [5]. It takes approximately 130 kilograms of lavender flowers to make 1 kilogram of lavender absolute [5]. Compared to lavender essential oil, it has a sweeter but less floral aroma. 

Recently, supercritical fluid extraction has revealed alternative “green” approaches for producing aroma products with various chemical compositions and sensorial properties.

At present, most reports have focused on the analysis of the chemical compositions of lavender essential oil [7,13,21,22,23,24,25,26,27,28] and lavender inflorescences’ volatile fraction [11,15,16,22,24,29,30,31], as well as the chemical contents of supercritical fluid extracts [32,33,34,35,36,37], with only a few investigations of the composition of the absolute found in the literature [31,38,39].

The study of the essential oil composition of 13 new Ukrainian cultivars of *L. angustifolia* by means of GC/MS was performed by Pokajewicz et al. [23]. Linalool and linalyl acetate were the principal constituents of all the samples, found in relatively broad ranges of 11.4–46.7% and 7.4–44.2%, respectively. It is worth mentioning that none of the studied samples met the requirements of Ph. Eur. and ISO 3515:2002. Küçük et al. [24] studied the volatile compounds of various *L. angustifolia* Mill. cultivars grown in Turkey. The main identified constituents were linalool, in the range of 31.9–50.0% and linalyl acetate, in the range of 15.4–42.0%.

A biochemical investigation of the essential oil from the leaves, flower buds and flowers of lavender was performed by Nurzynska-Wierdak and Zawislak [22]. The predominant compounds in the oil distilled from leaves were epi-*α*-cadinol (17.8%), cryptone (10.4%), 1,8-cineole (7.3%) and caryophyllene oxide (7.2%), and the predominant compounds of the oil distilled from flowers were linalyl acetate (22.3–32.1%) and linalool (23.9–29.9%).

Different extraction methods, including solid-phase trapping solvent extraction (SPTE), headspace solid-phase microextraction (HS-SPME), reduced pressure steam distillation (RPSD), and simultaneous steam distillation-solvent extraction (SDE) were used to comparatively study the volatile compounds of the Lavandula species. A total of 43 compounds were identified by GC/MS, with linalyl acetate and linalool being the predominant components of Hidcote lavender samples [29].

The volatile fractions of *L. angustifolia* inflorescences, extracted by different extraction techniques, were studied with respect to the yield and the chemical composition, revealing that water distillation (WD) produced the highest yields (1.2%) followed by water-steam distillation (WSD) (1.12%), solvent extraction (SE) (0.8%) and supercritical CO_2_ extraction (SCE) (0.5%). Linalyl acetate was found in higher amounts in the volatile fraction produced by SCE (51.8%), followed by SE and WSD (31.4% in each) and WD (26.8%) [30].

Da Porto et al. evaluate the flavour compounds of *Lavandula angustifolia* L., comparing three different extraction methods (hydrodistillation, supercritical CO_2_ extraction (SFE) and ultrasound-assisted extraction) regarding the use of extracts in food manufacturing [40]. The results show both quantitative and qualitative differences among the extracts, and the concentrations of linalool, camphor, linalyl acetate and (E)-caryophyllene were higher in the SFE extracts in comparison to the hydrodistilled oil, with linalool (43.30–45.78%) and linalyl acetate (17.91–21.00%) in SFE extracts, which also appeared in hydrodistilled oils at rates of 35.96–36.51% and 14.42–21.74%, respectively [41]. Chemical compositions of the tetrafluorethane subcritical extract from *L. angustifollia* were studied with respect to its chemical composition and antimicrobial activity by Atanasova et al. [32]. The main compounds found were linalool (32.48%), linalyl acetate (22.98%), borneol (5.12%), *cis*-linaloloxide (4.49%), (E)-*β*-farnesene (4.10%), lavandulol (4.22%) and β-caryophyllene (3.34%). The chemical profile and odor characteristics of lavender extracts and essential oil were studied by GC/MS, together with sensory evaluation [33]. Camphor and fenchon were found as major constituents in the volatile extract composition of *Lavandula stoechas* flowers obtained by hydrodistillation, subcritical water extraction and organic solvent extraction under ultrasonic irradiation, all of which were estimated by GC/MS [34]. A method of accelerated solvent extraction of lavender using butanol and dichloromethane as the solvent was studied in comparison with two conventional volatile isolation methods, including traditional steam distillation and Soxhlet extraction [35].

The supercritical fluid chromatography-atmospheric pressure photoionization–high-resolution mass spectrometry technique was applied for fingerprint analysis of different flower absolutes with uses in cosmetics and perfumery, including various *Jasminum and Narcissus* absolutes, as well as *L. angustifolia* absolutes from different suppliers and batches [38]. New constituents were discovered in a very unusual Spanish lavender: *Lavendula luisieri* (Rozeira) Riv. Mart. The essential oil and absolute of *L. luisieri* L., produced on an industrial scale, was studied, and many components unique in the plant kingdom with a cyclopentane skeleton, such as *α*-necrodyl acetate (20–25% of the oil) and *α*-necrodol, were identified by means of GC/MS [31].

Lavender concrete and absolute are important additional sources of compounds with valuable aromas and medicinal properties. It is necessary to mention that the characteristic fragrance and biological activities of lavender aroma products are determined by the composition of their volatile constituents. In this respect, the development of reliable analytical methods for origin assessment and quality assurance is of significant fundamental and practical interest. 

Therefore, the aim of the current study is to investigate in detail the chemical composition of lavender absolute industrially produced in Bulgaria from fresh lavender inflorescences and to compare it with the composition of absolute derived from exhausted lavender inflorescences. To the best of our knowledge, no such systematic comparative study creating chemical knowledge for origin assessment and quality assurance has been performed at present.

## 2. Materials and Methods

### 2.1. Samples

*Lavender absolute (LA) samples:* Seven industrial *LA* (*L. angustifolia* L.) samples were studied (2008-2017 harvest, marked as LA1–LA7, respectively), kindly provided by BulPhyto Oils Ltd. (Bulgarian producer of essential oils, absolute and floral waters, Sofia. Bulgaria). LA1-6 was extracted from Bulgarian lavender concrete derived from Lavandula angustifolia plants from the BulPhyto Oils fields (village of Zelenikovo area). Lavender concrete is extracted from fresh, unsteamed flower mass, harvested from the beginning of flowering to the stage of mass flowering at 40–80% blooming inflorescences and treated with a non-polar solvent (petroleum ether or n-hexane). It is a yellow-green to green-brown jelly-like mass that contains over 65% absolute. The absolute obtained from concrete is a yellowish-green, thin clear liquid with an aroma typical of lavender. 

One of the samples (LA7) is the so-called Lavera absolute—a dark green to brown viscous liquid with a lavender grassy scent with a hint of honey; it was obtained from Lavera concrete Lavera, which was derived from exhausted lavender inflorescences.

### 2.2. Methods

*Gas Chromatography*–*Mass Spectrometry (GC/MS)*: The GC/MS analysis was performed on an Agilent 7820A GC System Plus gas chromatograph coupled with 5977B Mass Selective detector and flame (ionization detector (Agilent Technologies, Palo Alto, CA, USA). Two fused silica capillary columns—an ultra-inert non-polar DB-5ms UI and a mid-polar DB-17HT (J&W Scientific, Folsom, CA, USA) with a 60 m column length, 0.25 mm i.d., and 0.25 μm film thickness, were used. The following gradient for the oven temperature was applied: from 60 °C (2.5 min held) to 155 °C at a rate of 2.5 °C/min and from 155 to 280 °C at a rate of 5 °C/min, with 10 min isotherm at the final temperature. Helium (99.999%) was used as the carrier gas at a constant flow rate of 0.8 mL/min. The split ratio was 1:125, the inlet temperature was set to 260 °C, and the transfer line temperature was 280 °C. The mass selective detector operated in electron impact ionization (EI) mode at 70 eV electron energy, the ion source temperature was set to 230 °C, and the quadrupole temperature was 150 °C. The scan mode was used for acquiring data with a mass range of 45–750 m/z.

*Gas Chromatography with Flame-Ionization Detector (GC-FID)*: The GC-FID analysis was performed on the same instrument under the same temperature gradient as described above. The system is equipped with a post-column flow splitter, allowing simultaneous analysis on both detectors. Instrument control and data collection were carried out using MassHunter Workstation Software (Revision B.06.07, Agilent Technologies).

*Identification, quantitative analysis and chemometrics*: The identification of the compounds was performed using commercial mass spectral libraries (NIST 14 Mass Spectral Library, Wiley 7th Mass Spectra Register) and retention times (linear retention indices, LRI). A homologous series of n-alkanes (C8–C40 Alkanes Calibration Standard, Supelco) was used for the experimental determination of the Kovats indices. In the cases of a lack of corresponding reference data, the structures were proposed based on their general fragmentation pattern and/or using reference literature mass spectra. The quantification of the main compounds was carried out by the internal normalization method with a response factor set equal to unity for all of the sample constituents. Despite not being considered a true quantification, a simple GC-FID percentage allows for comparison between the studied LA samples.

## 3. Results and Discussion

**Figure 1:**
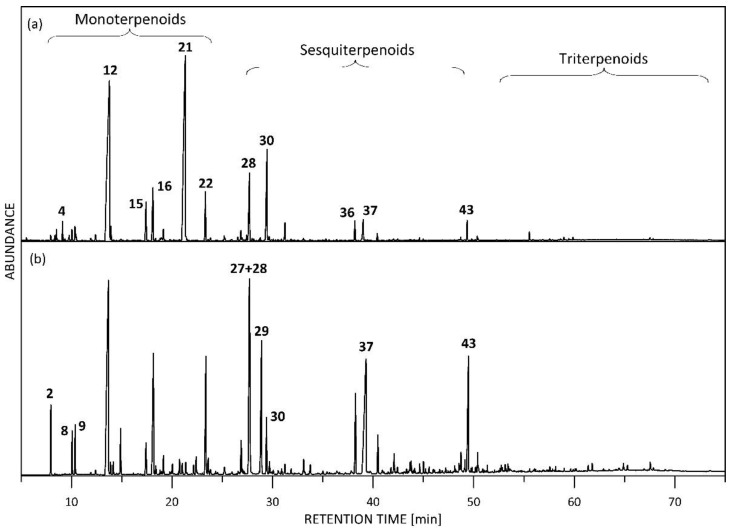
Representative GC/MS (TIC) chromatograms of LA samples: (**a**) LA6; (**b**) LA7. The main compounds found in Las are as follows: 2. *β*-myrcene, 4.3-octanol, 8. cis-*β*-ocimene, 9. *trans*-*β*-ocimene, 12. linalool, 15. lavandulol, 16. 4-terpineol, 21. linalyl acetate, 22. lavandulyl acetate, 27. nerolidol, 28. *trans*-*β*-caryophyllene, 29. geranyl acetate, 30. *trans-β*-farnesene, 6. caryophyllene oxide, 37. coumarine, 43. 7-methoxy-coumarine. The numbers of the compounds correspond to the numbering in Table 1.

**Figure 2:**
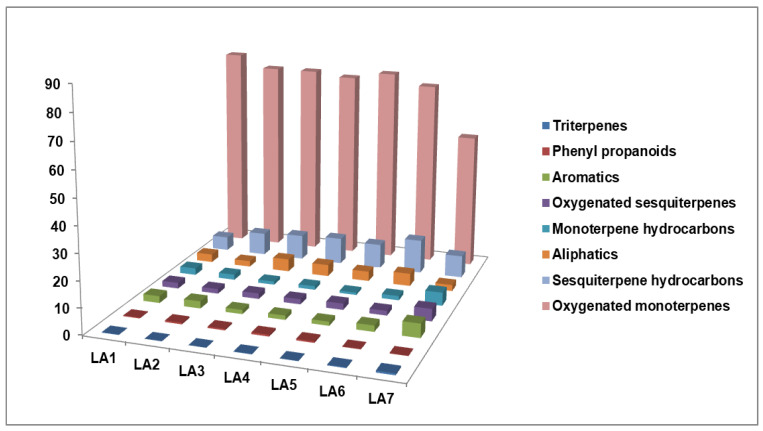
Main chemical class distribution in the studied LA samples.

As a result of the analysis, more than 150 compounds with concentrations higher than 0.01% were detected in LA samples, and 111 of them containing C2–C30 carbon atoms were identified by GC/MS and simultaneously quantified by GC-FID. The representative GC/MS chromatograms in the total ion current (TIC) mode of the LA samples are shown in Figure 1. The clearly distinguished chromatographic fingerprint of the LA7 sample can be seen. The quantitative content, as determined by GC-FID, of the compounds with concentration >0.1% is shown in Table 1, and the full list of the components identified in LAs is shown in Supplementary (Table S1). The compound distribution in different chemical classes is presented in Figure 2.

As seen from Table 1, the main constituents or LAs are representatives of the terpene compounds (with the dominating presence of monoterpenes, as well as sesqui- and triterpenoids as minor compounds) and aliphatics (hydrocarbons, ketones, esters, etc.). Fatty acids, higher alcohols and waxes were found as well.

It is worth mentioning that the studied absolutes show similar qualitative content with different quantitative characteristics. In general, according to the current results, the chemical profile of the studied LAs is close to the lavender essential oil and, with the exception of the LA7 sample, meets most of the requirements of the ISO 3515:2002 standard, also listed in Table 1.

### 3.1. Terpenoids

Terpenes and terpenoids are the main biosynthetic building blocks and important mediators of ecological interactions in plants. Of all the terpenoids, the mono- and sesquiterpenes are the main constituents of the essential oils and are most frequently studied because of their abundance in LO and their importance in ecology (plant–insect or plant–pest interaction) and in the flavor and fragrance industries [42]. 

### 3.2. Monoterpenes and Their Oxygenated Derivatives

*Monoterpene hydrocarbons:* Trans *β*-ocimene, known to have antibacterial and antifungal activity [43], is the most abundant monoterpene hydrocarbon in the LAs (0.30–1.40%), followed by its isomer *cis*-*β*-ocimene (0.37–0.71%). Their concentrations, though, are much lower in comparison with what is typical for *L. angustifolia* essential oil, derived by hydrodistillation according to ISO requirements.

*Monoterpene alcohols*: The acyclic monoterpene linalool is the main terpene alcohol, and, together with its ester linalyl acetate, they are the two main constituents in the LA samples. Various pharmacological properties are reported for linalool, such as antibacterial, antiviral, anti-inflammatory [43] and anxiolytic activity [3,44]. Linalool has been found to be the major pharmacologically active constituent involved in the anti-anxiety effect of lavender oil by Umezu et al. [3]. Strong antibacterial activity against Gram-positive and Gram-negative bacteria has been observed for (–)-linalool [43], which is the natural enantiomer in *L. angustifolia*, with >88% content in the Bulgarian LO [45]. Linalool content is in the range of 27.33–38.24% in the LA1–LA6 samples and 20.74% in the LA7 sample. The amount of linalool is very close to what is typical for the Bulgarian *L. angustifolia* essential oil. In the subcritical extract with tetrafluorethane derived from Bulgarian *L. angustifolia* cultivars, the amount of linalool was 32.48% [25]. The results for the essential oil from Lavandula species grown in Turkey are similar—31.9–50.0% for the lavender essential oil [17] and 34.0% in the oil from lavandin [32], while the linalool contents in the Turkish lavandin concrete and absolute were found to be much lower—17.7% in the concrete and 17.2% in the absolute, respectively [32].

The other monoterpene alcohols found in the LAs are 4-terpineol (3.05–6.50%), lavandulol (1.03–2.59%), 1,8-cineole (0.30–0.68%), and *α*-terpineol (0.30–0.58%). Citronellol, nerol and *trans*-carveol are also observed in low concentrations (<0.1%). It is interesting to mention that a mixture of monoterpenes containing terpinen-4-ol, α-terpineol and 1,8-cineole was active against Gram-positive and Gram-negative bacteria isolated from the skin, mouth and upper respiratory tract of humans [43]. On the other hand, terpinen-4-ol adds a bitter, herbal note to the lavender oil aroma; therefore, its content regarding requirements of the ISO standard has to be in the range of 2.0–5.0%. 

*Ketones*: Monoterpene ketones are observed in the LAs as minor components. Among them, camphor is the most important and most abundant compound with a concentration of 0.11–0.34%, followed by cryptone (0.36% in LA7; in other LAs, cryptone is co-eluted with linalyl acetate, and the quantitation is difficult) and piperitone in trace amounts (<0.05%).

*Esters*: Linalyl acetate is the most important ester, being responsible for the quality of the lavender aroma, and has been found in amounts ranging from 26.58 to 37.39% in the LA1-LA6 samples, while, surprisingly, it was not observed in LA7. It is worth noting that linalool and linalyl acetate show the same concentration range as in the LO in all LA samples, with the exception of the LA7 sample, where the linalool content is substantially lower, and linalyl acetate was not found. The linalyl content in the lavender subcritical extract was found to be lower—22.98% [25], while in the lavandine concrete and absolute, linalyl acetate was observed in much higher concentrations—46.6% and 45.0%, respectively [32].

Lavandulyl acetate shows a lower concentration in LA1–LA6 samples (1.47–2.94%), and its concentration in the LA7 sample is much higher—4.45%.

*Others*: Other monoterpenoids, such as furanoid linalool oxide (2-(5-Methyl-5-vinyltetrahydro-1-furyl)-2-propanol), are found as well. Despite being observed as a minor component (0.07–0.13%), linalool oxide adds an important note to the LA aroma.

### 3.3. Sesquiterpenes

*Sesquiterepene hydrocarbons*: *Trans*-*β*-farnesene (0.86–6.42%) and *trans*-*β*-caryophyllene (2.97–4.34%) are the main sesquiterepene hydrocarbons found in LAs, along with a variety of other sesquiterpenes presented as minor components, such as *α*-santolene (0.34–1.11%), germacrene-D (0.20–0.83%), α-bergamotene (0.20–0.37%), *γ*-cadinene (0.10–0.46%), *α*-humulene (0.14–0.40%), etc. In contrast to the observed monoterpenoids’ chemical profiles in the LAs, the sesquiterpene hydrocarbons are the more abundant chemical class compared to the sesquiterpene hydrocarbons. *Trans-β*-farnesene, although not monitored by ISO 3515:2002, is a very important microconstituent for the lavender essential oil flavour [20]. The content of these sesquiterpenes does not differ significantly from what is observed in the subcritical extract—4.10% for *β*-farnesene and 3.35% for *β*-caryophyllene [25].

*Oxygenated sesquiterpenes* found in LAs are representatives of the alcohols -*τ*-cadinol (0.21–1.04%), caryophyllenol (0.59% in LA7), *trans-β*-farnesol and its isomers, spathulenol, nerolidol (found in a concentration of 7.77% in LA7 only), *α*- and *β*-santalols in trace amounts, as well as ketones—muurol-5-en-4-one, hexahydrofarnesylacetone and oxides- caryophyllene oxide, observed in relatively high concentrations (0.88–1.59% in LA1–LA6 samples and 2.71% in LA7 sample), aromadendrene oxide, etc.

### 3.4. Triterpenes

Triterpenes are one of the most numerous and diverse groups of natural plant products, and they have a broad spectrum of biological activity. According to our results, the triterpene compounds are present in very low, trace amounts in all LA samples, and the following triterpenoids were tentatively identified: *α*- and *β*-amyrines, clionasterol (stigmast-5-en-3-ol(3-beta, 24S), and lupeol acetate.

### 3.5. Coumarins

*Coumarins* are a class of secondary metabolites (naturally occurring benzopyrone derivatives) with various pharmacological and biological effects. In the current study, coumarine (1.22–2.44%) and 7-metoxy coumarine (0.56–1.48%) were found in LA1–LA6 samples, while in LA7, both components were observed in much higher concentrations—10.9% and 3.83%, respectively.

### 3.6. Others

*Fatty acids, high alcohols and aldehydes, waxes*: These compounds, naturally presenting in lavender inflorescence, are normally not observed in the LO due to their low volatility, but they were found in relatively high amounts in the LAs. 

It is worth mentioning that the studied absolutes show similar qualitative contents with different quantitative characteristics. In general, according to the current results, the chemical profile of the studied LAs is close to the lavender essential oil and, with the exception of the LA7 sample, meets most of the requirements of the ISO 3515:2002 standard. In Table 2, the range of quantitative content variations between the LA (LA1–LA6) samples of the twelve compounds important for the quality and authenticity of Bulgarian LO compounds is shown, along with the limits of ISO 3515:2002 and European Pharmacopoeia, 8th Edition [46]. As seen from Table 2, the mean values of the two compounds most important for the quality of the LO, linalool and linalyl acetate, fit in the range of concentrations. The same is true for limonene, lavandulol and lavandulyl acetate, 1,8-cineole, 4-terpineol and 3-octanone. The mean values for *β*-ocimene and *α*-terpineol are much lower than in the ISO standard but still within the limits of Ph. Eur. According to Ph. Eur., lavender oil contains linalool (20.0–45.0%), linalyl acetate (25.0–46.0%), 1,8-cineole (<2.5%) and camphor (<1.2%).

## 4. Conclusions

Lavender’s absolute chemical composition is dominated by the presence of oxygenated monoterpenes, with linalool and linalyl acetate as the two principal components. Linalool was found in concentrations of 27.33–38.24% in the LA1–LA6 samples and 20.74% in the LA7 sample. The amount of linalyl acetate was in the range of 26.58 to 37.39% in the LA1–LA6 samples, while, surprisingly, it was not observed in LA7. With the exception of LA7, the observed chemical profile of the LAs was similar to the *L. angustifolia* essential oil chemical composition, fulfilling most of the requirements of the International Standard ISO 3515:2002.

## Figures and Tables

**Table 1:** Chemical composition (compounds with concentration >0.1%) of LA samples, as determined by GC/MS/FID on a DB-17HT column.

No	LRI_exp_	Compound	Lavender Absolute Rel. %,						
			LA1	LA2	LA3	LA4	LA5	LA6	LA7
	759	Ethanol	0.56	0.20	0.91	1.22	0.16	1.40	0.05
2.	968	*β*-Myrcene	0.19	0.26	0.19	0.21	0.15	0.31	2.15
3.	1051	1-Octen-3-ol	0.20	0.12	0.27	0.27	0.26	0.25	0.05
4.	1067	3-Octanol	0.15	0.13	0.53	0.52	0.51	0.51	n.d. ^1^
5.	1100	3-Octanone	0.59	0.43	1.15	1.15	1.07	0.97	n.d.
6.	1107	Limonene	0.12	0.08	0.08	0.08	0.07	0.09	n.d.
7.	1118	n-Hexyl acetate	0.30	0.29	0.41	0.41	0.38	0.24	n.d.
8.	1133	*trans-β*-Ocimene	0.71	0.61	0.52	0.61	0.37	0.63	1.42
9.	1125	*cis-β*-Ocimene	1.40	0.97	0.39	0.43	0.30	0.49	1.22
10.	1135	1,8-Cineole	0.68	0.74	0.36	0.35	0.34	0.30	n.d.
11.	1185	Linalool oxide	0.35	0.20	0.55	0.52	0.59	0.28	0.15
12.	1214	Linalool + 3-Octanol, acetate ^2^	31.67	27.33	38.12	37.30	38.24	36.12	20.74
13.	1221	1-Octen-3-yl acetate	0.74	0.60	0.63	0.54	0.63	0.68	0.39
14.	1244	n-Hexyl isobutyrate	0.07	0.08	0.15	0.15	0.15	0.10	1.32
15.	1299	Lavandulol	1.03	1.40	2.59	2.57	2.55	2.43	1.36
16.	1314	4-Terpineol	6.50	5.66	3.11	3.05	3.12	3.22	6.32
17.	1320	Camphor	0.23	0.34	0.11	0.11	0.11	0.12	0.30
18.	1330	Linalool oxide (2-(5-Methyl-5-vinyltetrahydro-1-furyl)-2-propanol)	0.07	n.d.	0.13	n.d.	0.12	0.10	0.12
19.	1332	Phenyl ethyl alcohol	0.25	0.61	0.56	0.68	0.57	0.13	0.10
20.	1337	*α*-Terpineol	0.30	0.39	0.57	0.56	0.58	0.55	0.53
21.	1383	Linalyl acetate + Cryptone ^2^	36.98	35.81	27.64	27.09	28.43	26.58	0.36 ^3^
22.	1427	Lavandulyl acetate	1.47	2.94	1.80	1.76	1.87	2.21	4.45
23.	1438	Cuminic aldehyde	0.11	0.15	0.12	0.12	0.12	0.14	0.19
24.	1458	*trans- α*-Bergamone (isomer) or *cis- α*-Bergamone	0.20	0.21	0.25	0.31	0.27	0.21	0.37
25.	1504	*α*-Santolene	0.34	0.41	0.39	0.39	0.40	0.45	1.11
26.	1506	p-Cymen-7-ol	0.08	0.10	0.13	0.14	0.13	0.12	0.16
27.	1522	Nerolidol	n.d.	n.d.	n.d.	n.d.	n.d.	n.d.	7.77
28.	1523	*trans-β*-Caryophyllene	3.01	3.41	2.97	3.05	2.78	4.34	3.54
29.	1551	Geranyl acetate	0.09	0.23	0.16	0.16	0.16	0.14	5.73
30.	1561	*trans-β*-Farnesene	0.86	3.26	5.06	5.20	5.11	6.42	1.93
31.	1567	*α*-Humulene	0.14	0.18	0.16	0.16	0.15	0.20	0.40
32.	1602	Germacrene D	0.21	0.28	0.28	0.33	0.20	0.83	0.34
33.	1616	*β*-Bisabolene	0.08	0.09	0.10	0.09	0.10	0.10	0.18
34.	1645	*γ*-Cadinene	0.16	0.25	0.10	0.10	0.10	0.14	0.46
35.	1650	*β*-Santalol	0.09	0.12	0.05	0.05	0.05	0.06	0.31
36.	1767	Caryophyllene oxide	1.23	1.03	1.36	1.25	1.59	0.88	2.71
37.	1787	Coumarine	1.25	2.44	1.25	1.23	1.22	1.33	10.9
38.	1825	*τ*-Cadinol	0.32	0.53	0.22	0.21	0.22	0.27	1.04
39.	1870	Alloaromadendrene oxide	0.27	0.20	0.20	0.19	0.22	0.10	n.d.
40.	1881	Farnesol 2	0.07	0.09	0.12	0.12	0.12	0.12	n.d.
41.	1921	*β*-Santalol (isomer)	0.07	0.09	0.05	0.04	0.05	0.05	0.27
42.	1934	Muurol-5-en-4-one	0.05	0.07	0.04	0.04	0.04	0.04	0.15
43.	2150	Coumarin, 7-methoxy-	0.89	1.48	0.56	0.57	0.57	0.60	3.83
44.	2491	10-Hydroxy-4-cadinene-3-one	0.16	0.25	0.10	0.10	0.10	0.10	0.56
45.	3347	*β*-Amyrin	n.d.	0.03	n.d.	0.09	0.03	0.03	0.10
46.	3393	Clionasterol (Stigmast-5-en-3-ol(3-beta,24S)	0.06	0.09	0.10	0.13	0.09	0.13	0.31
47.	3413	*α*-Amyrin	0.03	0.05	0.03	0.14	0.05	0.05	0.06
48.	3969	Lupeol acetate	0.06	0.06	0.05	n.d.	0.05	0.06	0.21
Total			97.43	94.28	96.05	94.96	95.70	97.31	95.25
Monoterpenes		Hydrocarbons	2.67	2.07	1.26	1.43	0.93	1.68	5.15
		Oxygenated	80.55	75.73	75.68	74.07	76.66	72.60	52.83
Sesquiterpenes		Hydrocarbons	5.51	8.90	9.77	10.38	9.64	13.29	8.71
		Oxygenated	2.29	1.82	2.18	2.03	2.47	1.82	4.78
Coumarins			2.15	3.92	1.81	1.80	1.79	1.93	14.73
Aromatics			2.68	2.81	1.61	1.67	1.58	2.41	5.41
Aliphatics			3.22	2.30	4.96	4.57	3.80	4.98	2.43

Remarks: ^1^ not detected, ^2^ co-eluting components, ^3^ the value is for cryptone in LA7.

**Table 2:** The quantitative content variations between the LA samples.

No	Compounds	LA Rel. %, as Determined by GC-FID				ISO 3515:2002	Ph. Eur. 8th Edition
		Min	Max	Mean	StDev		
1	3-Octanone	0.43	1.15	0.89	0.31	0.2–1.6	0.1-5.0
2	Limonene	0.07	0.12	0.09	0.02	<0.6	<1.0
3	*cis*-*β*-Ocimene	0.30	1.40	0.66	0.43	3.0–9.0	
4	*trans*-*β*-Ocimene	0.37	0.71	0.58	0.12	2.0–5.0	
5	1,8-Cineole	0.30	0.74	0.46	0.19	<2.0	<2.5
6	Linalool	27.33	38.24	34.80	4.39	22.0–34.0	20.0–45.0
7	Lavandulol	1.03	2.59	2.10	0.69	>0.3	>0.1
8	4-Terpineol	3.05	6.50	4.11	1.55	2.0–5.0	0.1–8.0
9	Camphor	0.11	0.34	0.17	0.10	<0.6	<1.2
10	*α*-Terpineol	0.30	0.58	0.49	0.12	0.8–2.0	<2.0
11	Linalyl acetate	26.58	36.98	30.42	4.68	30.0–42.0	25.0–46.0
12	Lavandulyl acetate	1.47	2.94	2.01	0.51	2.0–5.0	>0.2

## Data Availability

The data presented in this study are available in this article.

## Supplementary Materials

The following supporting information can be downloaded at: https://www.mdpi.com/article/10.3390/plants11223150/s1, Table S1: Chemical composition of LA samples, in rel. %, as determined by GC/MS/FID on a DB-17HT column.
