# Supplementary material for: Exploring the Chemical Composition of Bulgarian Lavender Absolute (Lavandula Angustifolia Mill.) by GC/MS and GC-FID
Source: Plants (Basel). 2022 Nov 17;11(22):3150. doi: 10.3390/plants11223150 (PMC9692913; doi:10.3390/plants11223150)
Supplement: Supplementary file 1 [file plants-11-03150-s001.zip › plants-2036249-supplementary/Table S1.pdf]

Table S1. Chemical composition of LA samples, in rel. %, as determined by GC/MS/FID on a DB-17HT column.

No	LRI $_{exp}$	Compound	Lavender absolute rel. %, as determined by GC-FID on DB-17HT							ISO 3515:2002
			LA1	LA2	LA3	LA4	LA5	LA6	LA7	
1.	759	Ethanol	0.56	0.20	0.91	1.22	0.16	1.40	0.05	
2.	765	Ethyl formate	0.12	0.08	0.09	0.09	0.05	0.06	0.06	
3.	771	Acetic acid	0.11	0.09	0.10	0.11	0.08	0.11	0.31	
4.	780	Ethyl acetate	0.09	0.05	0.06	0.08	0.02	0.02	0.04	
5.	815	Acetal	0.02	n.d.	0.01	0.02	n.d.	0.01	n.d.	
6.	839	n-Hexyl formate	0.08	0.05	0.19	0.19	0.17	0.14	n.d.	
7.	845	*α*-Pinene	0.05	0.05	0.03	0.03	0.02	0.03	n.d.	
8.	968	*β*-Myrcene	0.19	0.26	0.19	0.21	0.15	0.31	2.15	
9.	1051	1-Octen-3-ol	0.20	0.12	0.27	0.27	0.26	0.25	0.05	
10.	1067	3-Octanol	0.15	0.13	0.53	0.52	0.51	0.51	n.d.	
11.	1100	3-Octanone	0.59	0.43	1.15	1.15	1.07	0.97	n.d.	0.2-1.6
12.	1107	Limonene	0.12	0.08	0.08	0.08	0.07	0.09	n.d.	<0.6
13.	1118	n-Hexyl acetate	0.30	0.29	0.41	0.41	0.38	0.24	n.d.	
14.	1125	*cis-β*-Ocimene	1.40	0.97	0.39	0.43	0.30	0.49	1.22	3.0-9.0
15.	1133	*trans-β*-Ocimene	0.71	0.61	0.52	0.61	0.37	0.63	1.42	2.0-5.0
16.	1135	1,8-Cineole	0.68	0.74	0.36	0.35	0.34	0.30	n.d.	<2.0
17.	1151	*γ*-Terpinene	0.07	0.03	0.03	0.04	0.02	0.03	n.d.	
18.	1185	Linalool oxide	0.35	0.20	0.55	0.52	0.59	0.28	0.15	
19.	1214	Linalool+ 3-Octanol, acetate[1]	31.67	27.33	38.12	37.30	38.24	36.12	20.74	22.0-34.0
20.	1221	1-Octen-3-yl acetate	0.74	0.60	0.63	0.54	0.63	0.68	0.39	
21.	1227	neo-allo-Ocimene/Alloocomene	0.12	0.07	0.02	0.03	n.d.	0.03	0.36	
22.	1244	n-Hexyl isobutyrate	0.07	0.08	0.15	0.15	0.15	0.10	1.32	
23.	1257	1,3,5-Undecatriene	0.03	0.04	0.03	0.03	0.02	0.04	n.d.	
24.	1272	Cyclohexen-1-ol	0.05	0.04	0.04	0.04	0.04	0.03	0.05	
25.	1275	*trans, trans*-2,4-Octadecadienal	n.d.	n.d.	0.02	0.02	0.02	n.d.	n.d.	
26.	1299	Lavandulol	1.03	1.40	2.59	2.57	2.55	2.43	1.36	>0.3
27.	1314	4-Terpineol	6.50	5.66	3.11	3.05	3.12	3.22	6.32	2.0-5.0
28.	1320	Camphor	0.23	0.34	0.11	0.11	0.11	0.12	0.30	<0.6
29.	1330	Linalool oxide (2-(5-Methyl-5-vinyltetrahydro-1-furyl)-2-propanol)	0.07	n.d.	0.13	n.d.	0.12	0.10	0.12	
30.	1332	Phenyl ethyl alcohol	0.25	0.61	0.56	0.68	0.57	0.13	0.10	
31.	1337	*α*-Terpineol	0.30	0.39	0.57	0.56	0.58	0.55	0.53	0.8-2.0
32.	1351	Citronellol	0.06	0.09	0.05	0.06	0.06	0.08	0.08	
33.	1371	Isobornyl propionate	0.05	0.08	0.06	0.06	0.06	0.07	0.55	
34.	1377	p-Cymen-8-ol	n.d.	n.d.	n.d.	n.d.	n.d.	n.d.	0.44	
35.	1383	Linalyl acetate+Cryptone[1]	36.98	35.81	27.64	27.09	28.43	26.58	0.36[2]	30.0-42.0
36.	1389	t*rans*-Carveol	0.04	0.06	0.03	0.03	0.03	0.03	n.d.	
37.	1402	Geranyl ethyl ether 1 and 2	n.d.	n.d.	n.d.	n.d.	n.d.	n.d.	0.29	
38.	1403	Geranyl isobutyrate	n.d.	0.04	0.04	0.04	0.04	0.02	n.d.	
39.	1407	Geraniol	0.07	0.10	0.07	0.08	0.06	0.04	0.69	
40.	1410	exo-2-Hydroxycineole	0.02	0.04	0.03	0.04	0.04	0.03	0.06	
41.	1427	Lavandulyl acetate	1.47	2.94	1.80	1.76	1.87	2.21	4.45	2.0-5.0
42.	1433	*α*-Fenchyl acetate	0.11	0.17	0.05	0.06	0.06	0.06	0.47	
43.	1438	Cuminic aldehyde	0.11	0.15	0.12	0.12	0.12	0.14	0.19	
44.	1449	Piperitone	0.03	0.03	0.03	0.03	0.04	0.01	n.d.	
45.	1458	*trans- α*-Bergamone (isomer)	0.20	0.21	0.25	0.31	0.27	0.21	0.37	

		or *cis*- *α*-Bergamone							
46.	1470	*β*-Bourbonene	n.d.	n.d.	0.06	0.31	0.07	0.07	n.d.
47.	1480	Fenchone	0.02	0.04	n.d.	n.d.	0.03	0.02	n.d.
48.	1484	Zingberene	0.02	0.04	0.04	0.08	0.05	0.05	0.11
49.	1497	Hexenoic acid hexyl ester	0.07	0.10	0.17	0.18	0.17	0.13	0.09
50.	1504	*α*-Santolene	0.34	0.41	0.39	0.39	0.40	0.45	1.11
51.	1506	p-Cymen-7-ol	0.08	0.10	0.13	0.14	0.13	0.12	0.16
52.	1510	*trans*- *α*-Bergamotene	0.04	0.05	0.04	0.05	0.04	0.05	0.10
53.	1522	Nerolidol	n.d.	n.d.	n.d.	n.d.	n.d.	n.d.	7.77
54.	1523	*trans*-*β*-Caryophyllene	3.01	3.41	2.97	3.05	2.78	4.34	3.54
55.	1547	*α*-Amorphene	0.02	0.03	0.03	0.03	0.03	0.03	0.09
56.	1551	Geranyl acetate	0.09	0.23	0.16	0.16	0.16	0.14	5.73
57.	1557	Piperitenone	0.05	0.07	n.d.	n.d.	0.03	0.03	n.d.
58.	1561	*trans*-*β*-Farnesene	0.86	3.26	5.06	5.20	5.11	6.42	1.93
59.	1567	*α*-Humulene	0.14	0.18	0.16	0.16	0.15	0.20	0.40
60.	1576	Butyl benzoate	0.04	0.05	0.06	0.06	0.06	0.07	0.14
61.	1584	*β*-Farnesene isomer	0.05	0.06	0.07	0.07	0.06	0.07	0.17
62.	1602	Germacrene D	0.21	0.28	0.28	0.33	0.20	0.83	0.34
63.	1616	*β*-Bisabolene	0.08	0.09	0.10	0.09	0.10	0.10	0.18
64.	1625	*α*- Farnesene	n.d.	0.02	0.03	0.03	0.03	0.04	n.d.
65.	1645	*γ*-Cadinene	0.16	0.25	0.10	0.10	0.10	0.14	0.46
66.	1650	*β*-Santalol	0.09	0.12	0.05	0.05	0.05	0.06	0.31
67.	1704	Farnesol	0.02	0.05	0.06	0.05	0.06	0.04	0.03
68.	1715	Aromadendrene oxide	0.04	0.02	0.03	0.03	0.04	n.d.	n.d.
69.	1723	Caryophylene oxide	0.06	0.06	0.11	0.10	0.13	0.07	0.17
70.	1731	trans-Sesquisabinene hydrate	n.d.	n.d.	0.04	0.04	0.04	0.04	0.04
71.	1756	Spathulenol	0.04	0.05	0.04	0.04	0.03	0.03	n.d.
72.	1767	Caryophyllene oxide	1.23	1.03	1.36	1.25	1.59	0.88	2.71
73.	1787	Coumarine	1.25	2.44	1.25	1.23	1.22	1.33	10.9
74.	1793	Geranyl geraniol		0.02	0.05	0.04	0.04	0.05	n.d.
75.	1825	*τ*-Cadinol	0.32	0.53	0.22	0.21	0.22	0.27	1.04
76.	1837	Caryophylla-4(12),8(13)-dien-5-beta-ol	0.07	0.06	0.07	0.06	0.07	0.04	0.17
77.	1858	15-Hydroxy *α*- Muurolene)	0.03	0.03	0.05	0.05	0.06	0.04	0.17
78.	1870	Alloaromadendrene oxide	0.27	0.20	0.20	0.19	0.22	0.10	n.d.
79.	1873	Caryophyllenol	n.d.	n.d.	n.d.	n.d.	n.d.	n.d.	0.59
80.	1881	Farnesol 2	0.07	0.09	0.12	0.12	0.12	0.12	n.d.
81.	1918	*α*-Santalol	0.07	0.09	0.05	0.05	0.05	0.05	n.d.
82.	1921	*β*-Santalol (isomer)	0.07	0.09	0.05	0.04	0.05	0.05	0.27
83.	1934	Muurol-5-en-4-one	0.05	0.07	0.04	0.04	0.04	0.04	0.15
84.	1953	Hexahydrofarnesyl acetone	0.08	0.09	0.07	0.07	0.08	0.10	0.20
85.	1964	Geranyl linalool isomer B	n.d.	n.d.	n.d.	n.d.	n.d.	n.d.	0.13
86.	1970	Nerolidol	n.d.	n.d.	n.d.	n.d.	n.d.	n.d.	0.13
87.	1998	Farnesol isomer A	n.d.	n.d.	n.d.	n.d.	n.d.	n.d.	0.09
88.	2026	*trans*-Sesquisabinene hydrate	n.d.	n.d.	n.d.	n.d.	n.d.	n.d.	0.19
89.	2109	Hexadecanoic acid, ethyl ester	n.d.	n.d.	n.d.	n.d.	n.d.	n.d.	0.50
90.	2116	Muurola-4,10(14)-dien-1-beta-ol	0.06	0.07	0.05	0.12	0.05	0.05	n.d.
91.	2120	Benzyl benzoate	0.24	0.20	0.03	0.10	0.03	0.14	0.65
92.	2135	Geranyl-p-cymene	n.d.	n.d.	n.d.	n.d.	n.d.	n.d.	0.28
93.	2150	Coumarin, 7-methoxy-	0.89	1.48	0.56	0.57	0.57	0.60	3.83
94.	2225	9,12-Octadecadienoic acid, м	n.d.	n.d.	n.d.	n.d.	n.d.	n.d.	0.13
95.	2491	10-Hydroxy-4-cadinene-3-one	0.16	0.25	0.10	0.10	0.10	0.10	0.56
96.	2514	Labda-8(20),12,14-triene	n.d.	0.02	0.06	0.05	0.05	0.06	n.d.

97.	2571	1,6,10,14 Hexadecatetraen-3-ol, 3,7,11,15 tetramethyl-	n.d.	n.d.	n.d.	n.d.	n.d.	n.d.	0.17
98.	2589	9,12-Octadecadienoic acid, ethyl ester	n.d.	n.d.	n.d.	n.d.	n.d.	n.d.	0.12
99.	2661	9,12,15-Octadecatrienoic acid, ethyl ester	n.d.	n.d.	n.d.	n.d.	n.d.	n.d.	0.07
100	2679	Hexadecanoic acid, hexyl ester	0.02	n.d.	0.07	0.08	0.07	0.23	0.08
101	2691	Geranyl *α*-terpinene	n.d.	0.04	0.03	0.04	0.02	0.05	0.10
102	2718	9,12-Octadecadienoic acid, butyl ester	0.03	0.02	0.09	0.12	0.08	0.13	0.07
103	2762	9,12,15-Octadecatrienoic acid, butyl ester	n.d.	n.d.	0.02	0.03	0.02	0.07	n.d.
104	2796	Geranyl palmitate	0.02	0.02	0.09	0.09	0.09	0.11	0.07
105	2891	Geranyl oleate	n.d.	n.d.	n.d.	n.d.	n.d.	n.d.	0.18
106	2935	Neryl ester (WAX)	n.d.	n.d.	n.d.	n.d.	n.d.	n.d.	0.22
107	3130	Farnesyl acetate	n.d.	0.01	0.04	0.05	0.04	0.04	0.04
108	3347	*β*-Amyrin	n.d.	0.03	n.d.	0.09	0.03	0.03	0.10
109	3393	Clionasterol (Stigmast-5-en-3-ol(3-beta,24S)	0.06	0.09	0.10	0.13	0.09	0.13	0.31
110	3413	*α*-Amyrin	0.03	0.05	0.03	0.14	0.05	0.05	0.06
111	3969	Lupeol acetate	0.06	0.06	0.05	n.d.	0.05	0.06	0.21
Total			97.43	94.28	96.05	94.96	95.70	97.31	95.25
Monoterpenes		Hydrocarbons	2.67	2.07	1.26	1.43	0.93	1.68	5.15
		Oxygenated	80.55	75.73	75.68	74.07	76.66	72.60	52.83
Sesquiterpenes		Hydrocarbons	5.51	8.90	9.77	10.38	9.64	13.29	8.71
		Oxygenated	2.29	1.82	2.18	2.03	2.47	1.82	4.78
Coumarins			2.15	3.92	1.81	1.80	1.79	1.93	14.73
Aromatics			2.68	2.81	1.61	1.67	1.58	2.41	5.41
Aliphatics			3.22	2.30	4.96	4.57	3.80	4.98	2.43
